# Tracheotomy for Foreign Bodies in the Air Passages

**Published:** 1892-12

**Authors:** Willis F. Westmoreland

**Affiliations:** Atlanta, Ga.; Professor of Surgery in the Atlanta Medical College


### Southern Medical Record.

A MONTHLY JOURNAL OF MEDICINE AND SURGERY.

VOL. XXII. ATLANTA, GA., DECEMBER, 1892. No. 12

### Original Articles.

### TRACHEOTOMY FOR FOREIGN BODIES IN THE AIR PASSAGES.

BY WILLIS F. WESTMORELAND, M. D., ATLANTA, GA.
Professor of Surgery in the Atlanta Medical College.

My attention has been particularly called to this subject by the unusually large number of cases I have operated on in the last few years—I have made eighteen operations, and had under my personal supervision before I began to operate, a number more.

These cases, while not common, occur frequently enough to deserve more than passing notice from those who may be called upon to operate upon them frequently. This means the general practitioner as well as the surgeon.

The idea that no man, however old, or however great his observation, can possibly have much experience in this branch of surgery, and that any rule as to the handling of these cases must rest upon the united experience of the profession rather than upon any one's personal dicta, is to a *certain* extent true. But the usual way to study satisfactorily the collected experience of the profession, is that brought together in statistical tables. A great many of the tables are even worse than useless, for they lead to very erroneous deductions; deductions that have undoubtedly lead to the sacrifice of many lives. The largest number of cases I have seen collected in one article is one thousand; in this table the mortality from the operation is excessive enough to discourage the boldest operator, and the conclusions drawn by the author is that about as many or more recover from expulsion than from the operation. The value of these statistics are entirely lost from a scientific standpoint; they are collected by piece meal, and do not give the most important point-the length of time that has elapsed between the reception of the foreign body, and the time of the operation; nor can the importance of this point be too strongly emphasized, for in proportion to the time that has elasped before operation will the percentage of *mortality increase.*

From the study of such tables as give reliable *data,* and my own personal experience in the treatment of these cases, I have laid down this rule for my guidance, that the *certainty* of the *presence* of a *foriegn body* in the *air passages* makes an operation *immediately necessary,* and from this rule I make *absolutely no exception.*

A great variety of foreign bodies find their way into the air passages; the substance generally gets in during inhalation, or while laughing or coughing, and of course occurs, as a rule, among children. The symptoms exhibited by these cases vary according to the situation in which the foreign body is lodged, and its nature; it may lodge in the ventricles, or if light, as a seed, (and this is the most frequent substance that finds its way into the air passages), remain in the trachea and be carried up and down by the movements of respiration; or if it is heavy, as a coin, it is very apt to drop into one of the divisions of the bronchi, most frequently the right, and remain there. The *symptoms* will be more aggravated if it is irregular in shape or has sharp outlines.

The length of time that has elapsed since the occurrence of the accident also plays an important part in the seriousness of the symptoms. The symptoms for convenience may be divided into three stages: 1st. Obstruction immediately following the accident. 2d. Irritation produced by its presence. 3d. Inflammation coming on at a later period. If the patient survives the immediate results of obstruction, the stage of irritation follows. This is the time that the patient most frequently comes under the surgeon's notice. If nothing is done, the period of the inflammation gradually follows.

There is no marked difference in the symptoms of the three stages, and we will only speak of them in a general way. In all cases there is a feeling of intense suffocation, great difficulty of breathing, violent fits of spasmodic coughing, frequently attended with vomiting. The patient's face is haggard and wears a look of great anxiety. The symptoms subside, and are followed by a few moments of rest, but any movement on the part of the patient brings them on with renewed violence. Every time the foreign body is coughed up and strikes against the interior of the larnyx, it brings on the feeling of suffocation and an attack of spasmodic coughing, and if it happens to become impacted there, sudden death may result, apparently by the spasm induced. There have been a number of deaths from this before tracheotomy could be performed. One of my cases came very near dying in this way, and it convinced me that these cases do not bear waiting on, though the symptoms may be very mild. The case was that of a child two or three years old, in which the symptoms were unusually mild. The mother was sitting in the waiting room with the little one in her lap asleep. I opened the door going into the room when the youngster waked up and began to cough. In a moment he stopped breathing and threw himself back, making violent efforts at respiration. His face began to turn purple, and the mother began to shriek. I saw the situation was very critical, and that something had to be done quickly to dislodge the foreign body or the child would be dead in a few moments. I grabbed the child from the mother's arms, and rushed into the operating rooms, and in less time than it takes to write it, I had pushed an English catheter through the glottis, and dislodged the body, and relieved the spasm. In a few minutes the child was breathing all right again. It is not necessary to say that the child was operated on immediately, as they all will be in the future. I shall never forget the scene, though it lasted only a few moments. The look of anguish on the mother's face, or the look that she gave me, as she pressed, wildeyed, close to the lounge, or the child, when the body was displaced, losing its harrowing look of mortal agony, which was replaced by an expression that betokened relief and peace, and never will I again expose myself to a similar one, but shall prevent it by operating at once.

In making a diagnosis of these cases, I depend upon the history, that is, the sudden onset of symptoms, without previous history of any throat trouble, the fact that the patient thinks he has swallowed something, and, principally, upon hearing the whirr as the foreign body is carried up and down the trachea by the movement of respiration, and the click as it strikes against the walls of the trachea or larynx. In operating on these cases, it is not necessary to give an anæthetic, but merely a matter of option, for as soon as the incision is made through the skin there is no pain; however, I always give it, as it keeps the patient perfectly quiet. Then by careful dissection, layer by layer, I go down to the trachea. When this is reached, I stop all hemorrhage, ligating *every vessel*, whether vein or artery, that bleeds, with cat gut, before opening the trachea; when this is opened, I put in a pair of tracheal forceps to keep it open.

Now, a few words by way of parenthesis. In all the cases I have had, tracheotomy has been the operation of selection. The opening in the trachea beginning with the first ring and continued downwards until a sufficiently large opening is made; when I have met with anomalies in regard to the blood vessels, and it has been necessary to cut them, I have caught them between two artery forceps, cut and ligated before proceeding further, thus keeping the field free from blood. If the foreign body is not immediately blown out, I allow the patient to come out from under ether, and then excite enough irritation by movement of the tracheal forceps to cause coughing, when the body generally comes out. If it does not, I keep the tracheal wound open until it does. Now, in keeping the wound open for the expulsion of the foreign body, or the mucus afterwards, I have never in a single instance used the tracheotomy tubes, but as I pointed out a number of years ago, have passed a piece of strong silk thread six inches in length through each side of the tracheal incision, and knotting together both ends of each piece of thread to prevent its slipping out. This makes the best of retractors, without the irritation or slipping you so frequently have at an important moment, from any of the instruments that can be used. The threads can be left in any number of days without irritation until the body is expelled. In one case I left them in four days. It is not necessary to pass the threads through the skin or muscles, as they will retract sufficiently without any assistance. The trachea-tubes are dangerous, not only on account of increasing the inflammatory trouble that may be present by their irritation, but also by acting as an obstruction to the expulsion of the foreign body, or mucus, if the body be thick. Then, there is the additional danger of their stopping up, and irritation of removing and reinserting when they have to be cleaned. I have put my trachea-tubes in a box and marked on it, "Let alone; dangerous."

At this stage of the operation, (that is, after the trachea is opened), I divide these cases into three different classes so far as their further treatment is concerned. In the first two classes, the foreign body is supposed to have escaped at the time of operation.

Class 1. Are those cases that can be closed immediately after the removal of the foreign body. The patient is seen and the operation made shortly after the accident. The foreign body comes away promptly, the mucus that has accumulated in the trachea as the result of the slight irritation is coughed out, and the trachea is ready to be closed in a few minutes. I close the wound with three separate layers of continuous cat-gut sutures, the first through the membrane enclosing the trachea, also the isthmus of the thymus gland, if it has been cut; the second bringing the muscles together, and the third the skin. Sometimes, when I think it is necessary, I place a few strands of cat-gut at the lowest angle of the wound for drainage, and then dress antiseptically. In these cases union is secured by first intention, with no perceptible scar.

Class 2. Are those that are operated on after considerable irritation or inflammation has been set up, either by the length of time the foreign body has remained or on account of its roughness. In these cases, though the foreign body may come away promptly, it is not safe to close the wound on account of the immense accumulation of mucus. I pass the silk threads through the edges of the tracheal incision and keep the trachea open until it is comparatively clear, then allow the edges of the trachea to come together as close as they will. I place over the wound a handful of loose bi-chloride gauze and hold in place with a lightly applied bandage. This dressing will protect the trachea and wound, but will still admit of the mucus that does not easily escape through the mouth, being coughed through the opening into the dressing.

I have found that where the wound is protected from the air, etc., in this way, the inflammatory symptoms subside much more rapidly and the silk threads can be removed and the wound closed in a short time in the manner already mentioned.

Class 3. Are where there is not only the results of the inflammatory trouble, but where the foreign body does not come away at the time of operation.

When the foreign body does not come away, after waiting a short time, after the trachea has been opened, instead of the usual plans of trying to remove it by instrumentation, I simply place a loose dressing of gauze over the wound, and leave it to the effects of nature, feeling assured that she will expell it with less irritation than mine would cause. I do not feel any uneasiness for the threads in each side of the trachea in the manner I have already suggested, gives yourself or the nurse perfect control of it.

I have waited in this manner in four cases, varying from six hours to four days, with perfect success; and expect to continue the same plan in the future, feeling assured that the mortality in these cases is frequently increased by the meddlesome interference of the surgeon.

In these cases after the foreign body is expelled the wound can be closed in the manner already suggested.

---

A CONDENSED MILKMAID.—Wee Miss.—"Mamma, may'nt I take the part of a milkmaid at the fancy ball?"

Mamma.—"You are too little."

Wee Miss.—"Well, I can be a condensed milkmaid."—*Good News.*

